# Regional Lassa virus lineages select for divergent MHC-I repertoires in *Mastomys natalensis* rodents

**DOI:** 10.1371/journal.ppat.1014121

**Published:** 2026-04-17

**Authors:** Ayodeji Olayemi, Jan Sarapak, Kerstin Wilhelm, Stephan Günther, Simone Sommer, Elisabeth Fichet-Calvet, Dominik Werner Melville

**Affiliations:** 1 Natural History Museum, Obafemi Awolowo University, Ile Ife, Osun, Nigeria; 2 Zoonoses Control Research Group, Bernhard Nocht Institute for Tropical Medicine, Hamburg, Germany; 3 Institute of Evolutionary Ecology and Conservation Genomics, University of Ulm, Ulm, Germany; 4 Department of Virology, Bernhard Nocht Institute for Tropical Medicine, Hamburg, Germany; UTMB: The University of Texas Medical Branch at Galveston, UNITED STATES OF AMERICA

## Abstract

Identifying genes under local adaptation is an essential step to understand the mechanisms of adaptive evolution. Pathogen-mediated selection is expected to enhance host fitness by favouring resistance to locally prevalent pathogens. However, such pathogen-driven adaptation has been documented in only a few natural systems. Here, we sequenced the Major Histocompatibility Complex Class I region (MHC-I) of 739 *Mastomys natalensis* captured in Guinea and Nigeria, where the rodent is reservoir to two distinct Lassa virus (LASV) lineages. As predicted, the MHC-I profiles of the two countries, while showing overlap, did not cluster together. Moreover, different MHC-I alleles were associated with active or past infection measured as PCR-positive or IgG-positive, respectively, in each population. MHC-I allele ManaMHC-I*017 showed a diametric response, with individuals carrying this allele less likely to be found with an ongoing LASV infection in Guinea while more likely in Nigeria. Similarly, individuals with ManaMHC-I*069 were less likely to have a positive antibody test in Guinea but the same allele had little effect on IgG detection in Nigeria, suggesting that an individual’s fitness depends on its immunogenetic repertoire. Together, these findings encapsulate a genetically characterised case of local adaptation in a wild virus–rodent system. Moreover, we hypothesise that aside from differences in virus diversity, genetic variation within regional LASV lineages contributes to the marked differences in host immunogenetic diversity.

## Author summary

The Lassa virus (LASV), which occurs in West Africa and consists of up to seven lineages, has the Multimammate mouse *Mastomys natalensis* as its major natural reservoir and kills thousands annually when it spills over into humans. Our results indicate that MHC class I, an important immunogenetic marker, is structured differently in *M. natalensis* populations inhabiting regions endemic for LASV lineage II in southwestern Nigeria and lineage IV in Guinea. MHC-I constitution between these regions diverges in overall allele composition and also in the specific alleles associated with susceptibility (indicated by active, ongoing infections) and resistance (inferred from cleared infections showing antibodies) to LASV. Additionally, the higher average number of MHC class I alleles observed in *M. natalensis* individuals from Guinea appeared to correspond to the greater genetic diversity of LASV lineage IV, in contrast to the lower average number of MHC class I alleles associated with LASV lineage II in the sampled localities. These signatures of local MHC-I adaptation to LASV offer further insight into immunology and infection dynamics of the virus in *M. natalensis*. This insight is also integral to measures such as zoonotic risk mapping and vaccine design to control the virus in its natural rodent host.

## 1. Introduction

**Fig 1:**
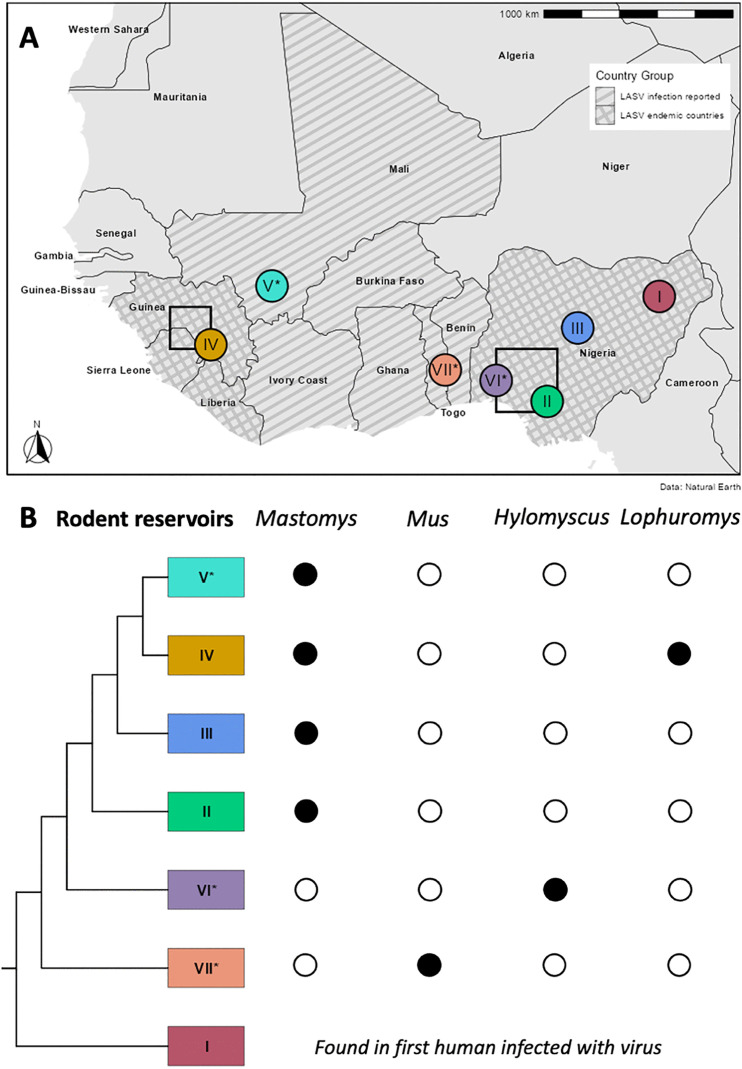
Distribution of LASV lineages and rodent reservoirs in West Africa. **A)** Distribution of sporadic and endemic hotspots for the seven recognised LASV lineages and B) their phylogenetic relationship and known rodent reservoirs. Black rectangles surround areas containing field sites within Guinea and Nigeria, respectively. Asterisks mark LASV lineages that break out sporadically in humans. The basemap was downloaded from Natural Earth using the R libraries *rnaturalearth* and *rnaturalearthdata*, and adapted directly in the R environment.

Lassa virus (LASV), a zoonotic mammarenavirus endemic to West Africa, causes a severe viral haemorrhagic disease and infects nearly 900,000 people annually, with approximately 18,000 deaths from Lassa fever [1,2]. Rodents are the natural LASV reservoir, particularly the Natal multimammate mouse (*Mastomys natalensis*), with human infection thought to occur mainly through contact with contaminated faeces or urine [3,4]. Despite the broad distribution of *M. natalensis* across sub-Saharan Africa, LASV seems phylogeographically tied to the mitochondrial clade A-I of *M. natalensis* inhabiting West Africa [5]. However, the virus diversified across regional rodent populations, resulting in seven genetically distinct virus lineages [6–9] (Fig 1). In response, hosts are predicted to adapt to local virus lineages, though data on (immuno-)genetic differences among subpopulations of *M. natalensis to test this hypothesis is missing so far*.

The Major Histocompatibility Complex (MHC) is the best understood genetic basis of pathogen resistance [10,11]. The MHC is a group of genes in jawed vertebrates that code for cell-surface glycoproteins which recognize and bind to antigens from pathogens, setting off the immune response [12,13]. Class I MHC (MHC-I) mainly binds to intracellular microbes such as viruses and protozoa, while MHC-II apprehends extracellular parasites [14]. A hallmark of the MHC region is its extreme gene polymorphism, which enables the host to detect a wide variety of pathogens and keep up with fast-evolving pathogens [15,16], such as RNA viruses and bacteria [17–19], as well as mediate the relationship with the host’s symbiotic microbes [20,21]. The major mechanisms that maintain this heterogeneity include the heterozygote advantage, conferring greater protection on individuals possessing a more divergent composition of alleles; the rare allele advantage, favouring individuals with uncommon alleles; and fluctuating selection over time and space, which often equates to a MHC repertoire adapted to the host’s local pathogen pool [22–27].

The MHC-I region in mammals and particularly rodents is notoriously understudied owing to its exceptionally high diversity when compared with the MHC-II region [28–36]. The MHC-I of *M. natalensis* from southwestern Nigeria was recently sequenced for the first time, revealing considerable diversity of 193 alleles among 189 individuals, with an average of 28 alleles per individual; variability enabled by MHC-I duplication to an extent of 22 loci [37]. MHC-I composition differed between endemic and non-endemic localities, with a larger proportion of rare alleles described in the endemic area, where LASV lineage II circulates. Furthermore, allele ManaMHC-I*006 was found to be associated with resistance to LASV (i.e., prompt clearance and antibody response) while alleles ManaMHC-I*008 and ManaMHC-I*021 were linked to active infection, indicating susceptibility [37]. Yet, the scope of the work was limited to two capture sites and relatively low sample sizes, given the immense diversity at the *M. natalensis* MHC-I [38]. Moreover, the diversification of LASV into regional lineages begs the question as to whether *M. natalensis* adapts immunogenetically to divergent LASV lineages.

This study takes advantage of the distinct LASV lineages found along the West African range of *M. natalensis* to elucidate whether the rodent’s MHC-I repertoire shows signatures of local adaptation. In southern Nigeria LASV lineage II is prevalent whereas lineage IV is present in Guinea [6]. In terms of geographical scope and number of virus-positive rodents detected to date, these two lineages represent the most consequential among the seven currently reported [3,39–44]. Yet, with regard to virus-rodent host interaction, certain distinctions exist. For instance, greater LASV prevalence in *M. natalensis* has been recorded for lineage II, while a relatively higher abundance of this rodent is common in localities where lineage IV circulates [44]. We predicted that the distinct LASV lineages (and other viruses likely dissimilar between Guinea and Nigeria) selected for divergent MHC-I composition, although we also expect to see some shared alleles, albeit likely at different frequencies. Furthermore, we expect that particular MHC-I alleles associated with susceptibility (consistent with active, ongoing infections that are PCR-positive, as defined in Olayemi et al. (2024) [37]) and resistance (congruent with promptly cleared infections, showing a residual IgG signal), will differ owing to adaptation to distinct LASV lineages. Locally divergent effects of MHC-I alleles may argue for spatially fluctuating pathogen-mediated selection. Our findings suggest that the MHC-I repertoire of *M. natalensis* indeed diverged to meet challenges by local virus lineages.

## 2. Methods

### 2.1. Ethics statement

This study adhered to ethical guidelines for the use of animal specimens. *Mastomys* rodent samples were processed as part of ongoing research efforts into the genetic diversity of MHC-I and its association with LASV infection. Ethical approval for the Nigerian samples was obtained from the Health Research Ethics Committee of the Irrua Specialist Teaching Hospital in Edo State, Nigeria (ISTH/HREC/2017/1019/28). For the Guinean samples, ethical clearance was obtained from the “Comité National d’Ethique pour la Recherche en Santé” (12/CNERS/12). Rodents were euthanized with isoflurane prior to tissue collection. The procedures followed strict safety protocols during collection, necropsy, and sample processing.

### 2.2. Sample collection and study areas

We compiled genetic and infection data from 191 and 344 *M. natalensis* sampled in Nigeria and Guinea, respectively, and added published data from the 204 samples collected in Nigeria [37]. Our *M. natalensis* collection from various sites fall within the distribution range of mitochondrial lineage A-I, though given the limited home range of *M. natalensis* these represent independent populations [5,45,46]. Trapping focused on locations associated with LASV lineage II infections

in Nigeria and LASV lineage IV infections in Guinea (Fig 1A). In Nigeria, the localities Abagboro, Ebudin, Ekpoma, Ifon, Okeluse, Okhuesan, and Owo were sampled. Guinean samples originated from Damania, Sokourala, Sonkonia, Tambaya (Table 1). Endemic locations are considered hot spots of LASV, though LASV may still occur sporadically in non-endemic regions at low prevalence.

**Table 1:** Endemic and non-endemic sampling locations including their geographic coordinates, the number of captures and the final number of uninfected, LASV positive and IgG positive samples with unambiguous MHC-I sequencing information used for hypothesis testing.

Location	Latitude	Longitude	Capture size	LASV Endemicity	MHC-typed sample sizes (uninfected/LASV + /IgG + /both)
**a) Guinea**					
Damania	9° 80’ 66.99’‘ N	10° 86’ 33.39’‘ W	110	Endemic	(49/17/32/2)
Sokourala	10° 05’ 64.34’‘ N	10° 66’ 52.59’‘ W	79	Endemic	(34/12/19/5)
Sonkonia	9° 91’ 30.43’‘ N	10° 79’ 77.66’‘ W	105	Endemic	(76/4/19/-)
Tambaya	10° 17’ 0“N	14° 7’ 0“W	50	Non-endemic	(45/-/1/-)
**b) Nigeria**					
Abagboro	7° 32’ 38.0“ N	4° 30’ 47.2“E	81	Non-endemic	(76/-/1/-)
Ebudin	6° 35’ 48.4“N	6° 10’ 53.3“E	123	Endemic	(54/9/25/-)
Ekpoma	6° 44’ 29.1“ N	6° 06’ 17.6“E	115	Endemic	(59/20/28/2)
Ifon	6° 55’ 30.6“ N	5° 46’ 34.5“E	16	Non-endemic	(10/-/6/-)
Okeluse	6° 47’ 1.0“ N	5° 35’ 10.9“E	8	Non-endemic	(4/1/3/-)
Okhuesan	6° 36’ 40.2“ N	6° 24’ 15.2“E	27	Non-endemic	(21/-/5/1)
Owo	7° 12’ 28.2“ N	5° 35’ 04.2“E	25	Endemic	(14/11/-/-)

Two trapping strategies were employed: For rural localities, trapping was conducted indoors along a single transect through each village, with additional transects at the village edges for outdoor captures. In urban localities, trapping took place indoors and outdoors at specific addresses spread across the town, including both locations with and without a history of Lassa fever cases in humans [47]. All villages in Guinea and the Nigerian localities of Abagboro, Ebudin Okeluse, and Okhuesan were considered as rural and sampled according to the first strategy. The larger localities of Ekpoma, Ifon, and Owo were sampled following the urban trapping strategy. During rodent collection in the field, individual weight, sex, and eye lens weight were noted, and individuals were identified down to species level, if possible. Thereafter *M. natalensis* identification was confirmed via mitochondrial cytochrome b sequencing, as described previously [47].

### 2.3. Lassa virus detection

At least seven distinct LASV lineages have been identified in West Africa and can be differentiated by phylogenetic analysis (e.g., [48]) of PCR-generated virus genetic sequences [49,50]. LASV lineage I, now possibly extinct, was isolated from among the first patients diagnosed in Lassa village in north western Nigeria, from which the disease’s name is coined [51]. Lineage II occurs in southern Nigeria and is hosted by *Mastomys natalensis*; and to a lesser extent, the Guinea multimammate mouse *M. erythroleucus* [39]. Lineage III exists in central Nigeria and has been detected in *M. erythroleucus* [43]. Lineage IV circulates across the Mano River Union countries (Guinea, Liberia & Sierra Leone), is hosted by *M. natalensis*, and to a lesser extent, by *M. erythroleucus* and the Brush furred rat *Lophuromys sikapusi* [40,43,52]. Lineage V, hosted by *M. natalensis*, occurs in southern Mali and northern Côte d’Ivoire [53,54]. Lineage VI was found in the African wood mouse *Hylomyscus pamfi* within southwestern Nigeria [43], and lineage VII in the Pygmy mouse *Mus baoulei* within Benin and Ghana [9,55].

In this study, active LASV infections were detected using PCR [3,39,49,50], while previous infections were identified through an Immunofluorescence Assay targeting IgG antibodies [3,44,47]. LASV genetic sequences obtained from PCR-positive *Mastomys* from our collection within Nigeria were phylogenetically classified as lineage II [39] and those from Guinea as lineage IV [3]. LASV diversity between these lineages in our data set, measured in terms of percentage similarity among glycoprotein sequences, was compared using the MacVector software (MacVector Inc, version 18.8.2).

### 2.4. MHC-I characterisation

For MHC-I characterisation, kidneys were preserved in ethanol and used for DNA extraction, performed with the DNeasy Blood & Tissue Kit (Qiagen, Germany), following the manufacturer’s protocol. DNA quality and quantity were assessed prior to sequencing excluding samples with too low concentrations for sequencing. MHC-I sequencing followed the protocol outlined in the prior study, with identical primers (Ma16F and Ma257R) and PCR conditions [37]. Both Nigerian and Guinean samples underwent the same two-step PCR process using the Fluidigm Access Array System for Illumina Sequencing Systems. Negative PCR controls were included in all batches to monitor contamination (n = 15). Additionally, 216 replicates were run to assess repeatability.

Illumina MiSeq sequencing was performed with a V2 reagent kit (2x250 cycles). Sequencing produced a total of 19,412,272 and 41,860,813 raw reads for Guinea and Nigeria, respectively. The sequences were analyzed using the “Allele Calling Procedure for Illumina Amplicon Sequencing” pipeline (ACACIA), which improves allele calling and data filtering [56]. Nigerian and Guinean samples were run separately. To account for the exceptionally high genetic diversity in MHC sequences [57], we raised the entropy threshold from 0.2 to 0.35 and required a minimum of 20 reads for a sequence to be considered an MHC allele, as done previously for this species [37]. These stringent filtering steps remove low-quality reads, chimeras, and artefactual sequences, and only samples passing these quality checks were retained for downstream analysis. The allele sequences were matched to previous records [37] and named accordingly unless the sequence was new.

Additionally, we filtered for unreliably genotyped MHC alleles [58,59]. First, we computed the frequency of each allele both within and across amplicons as the amount of sequencing reads retrieved for the particular allele averaged across all amplicons (i.e., mean per amplicon frequency; ‘MPAF’). We then retained only alleles passing the threshold of 0.05 MPAF and with proportional abundance of at least 1% (as to decrease chance of including chimeras that often occur at low frequencies compared with their original sequences) [60]. Secondly, if technical replicates differed from one another, we selected technical replicates for analysis by prioritizing replicates with both the highest total read count and the greatest number of distinct alleles. If no single replicate met both criteria, the entire sample was excluded from further analysis. In total, 89.4% of replicates were repeatable, similar to the 93.8% found previously in Nigeria alone [37]. After these quality checks, we arrived at reliable MHC-I data for 315 Guinean and 350 Nigerian samples (Table 1).

### 2.5. MHC-I supertyping

Positive selection across codon sites was assessed using CodeML within the PamlX graphical user interface [61], resulting in the identification of 14 positively selected sites (PSSs). To characterize the physicochemical binding properties of each amino acid at these PSSs using z-values and then cluster alleles with potentially similar antigen-binding profiles into supertypes we utilized MHCtools [62]. MHCtools determined the optimal number of clusters through high-throughput calculations, including distance matrix creation and BootKMeans clustering (permutations = 1000), with the optimal k selected based on statistical metrics such as Mean Total Within-Cluster Sum of Squares Residuals and Akaike and Bayesian Information Criterion (AIC/BIC) values. Following this, DAPC clustering was employed to assign alleles into the identified supertypes, summarizing those with similar antigen-binding characteristics [63]. A total of 10 alleles were classified as admixed, which we defined as alleles with single-cluster membership probability < 85%, and, thus, excluded from supertyping.

### 2.6. Statistical analyses

To test whether the composition of MHC-I alleles and supertypes differed between countries and across localities within countries we used a permutational analysis of variance (1000 permutations), computed with the *adonis*() function in the ‘vegan’ package [64]. Allele compositions were computed as Manhattan distance. We then followed previous approaches [30,37,65], to first probabilistically determine associations between individual MHC-I alleles, supertypes, and active (i.e., LASV+) or previous LASV infection (i.e., IgG+) using the *cooccur*() function from the ‘cooccur’ package [66]. We only included MHC alleles and supertypes present in more than 10% of individuals, and in cases where alleles were correlated with each other (r ≥ 0.8) we retained only the allele at higher frequency for further analyses [67]. To identify candidate alleles/STs, the co-occurrence analysis was completed only on data from LASV endemic villages. This approach allowed us to then test positively (i.e., more commonly found in connection with LASV + /IgG-, implying susceptibility) or negatively (i.e., more commonly found in connection with LASV-/IgG + , implying resistance) associated candidate alleles with a generalized linear mixed effect model for their likelihood to determine active or previous LASV infections across the entire dataset. Besides individual allelic diversity, which may play a role in defending against a variety of pathogens [16], the model included additional biologically meaningful covariates, such as sex, country (as surrogate for LASV lineage), and eye lens weight (as surrogate for rodent age) [68,69] as well as sampling year as random effect correcting for temporal variance. To assess sign changes in associations among rodents co-evolved with regional lineages, we tested for allele-by-country interactions. Model selection followed the information-theoretic approach employing the function *dredge()* from the MuMin package to rank competing models using Akaike’s Information Criterion [70]. Among the competitive model structures (deltaAIC < 2.0) the most complex was reported. Lastly, only p-values corrected for false-discovery rate are reported [71].

## 3. Results

### 3.1. MHC-I diversity in Nigerian and Guinean *Mastomys natalensis*

**Fig 2:**
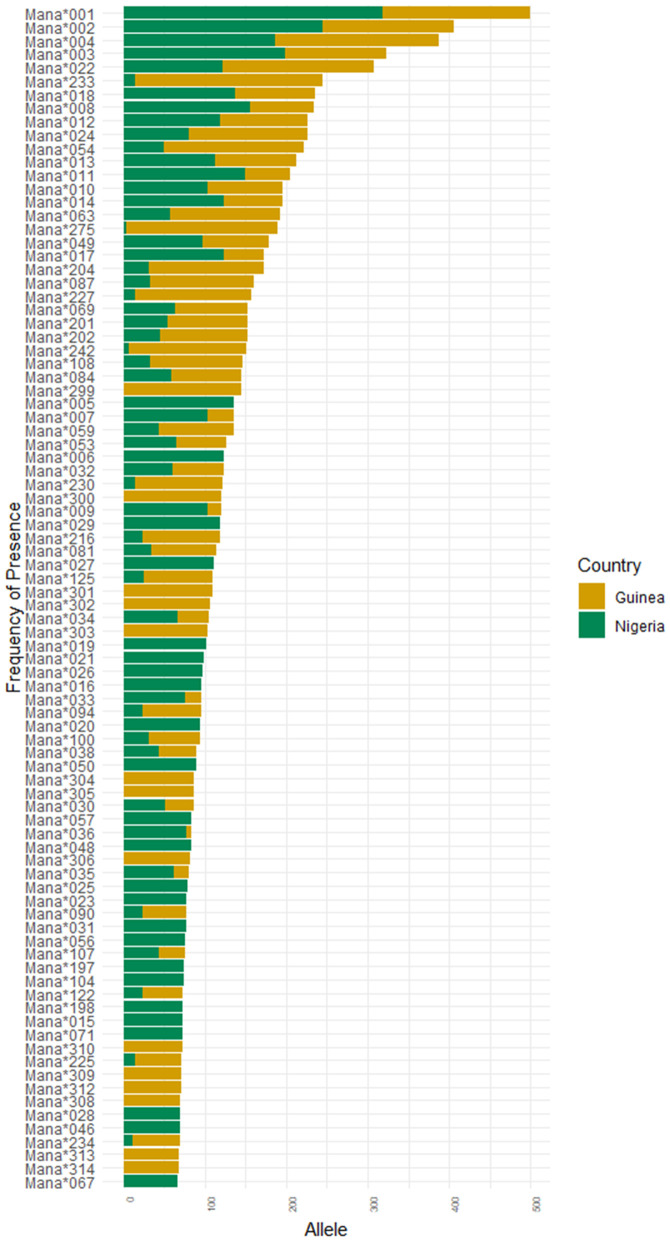
MHC-I allele frequencies of *M. natalensis* captured in Guinea (yellow) or Nigeria (green). Only alleles with frequencies > 10% shown.

After filtering for quality, we detected a total of 393 MHC-I alleles in 665 out of 739 *M. natalensis* rodents captured in Guinea and Nigeria between 2011 and 2019 (Table 1, S1 Fig.). The genetic sequence of each MHC-I allele are listed in S1 Appendix. Fig 2 shows the frequency of the more common alleles.

We found a maximum number of 52 alleles in at least one individual, suggesting at least 26 loci. The average number of MHC-I alleles per individual differed between Guinea and Nigeria (Guinea = 30.0 ± 7.1 SD; Nigeria = 27.6 ± 7.1 SD; t-test: p-value<0.001), with increased allele richness in Guinea consistent with higher LASV diversity (i.e., lower virus sequence similarity) observed for this country within our *M. natalensis* collection (Table 2).

**Table 2:** LASV sequence diversity and MHC-I allele richness between Guinea and Nigeria.

	Guinea *M. natalensis* N = 315	Nigeria *M. natalensis* N = 350
MHC-I diversity		
Average number of alleles per individual Mean ± SD	30.01 ± 7.11	27.62 ± 7.06
LASV diversity	38 glycoprotein virus sequences, lineage IV (Mariën et al. 2020)	42 glycoprotein virus sequences, lineage II (Adesina et al. 2023)
% Nucleotide sequence similarity	92.5 ± 2.8% [87.6-100]	93.3 ± 2.2% [89.2-100]
% Amino acid sequence similarity	97.4 ± 1.1% [94.6-100]	98.3 ± 0.8% [95.2-100]

**Fig 3:**
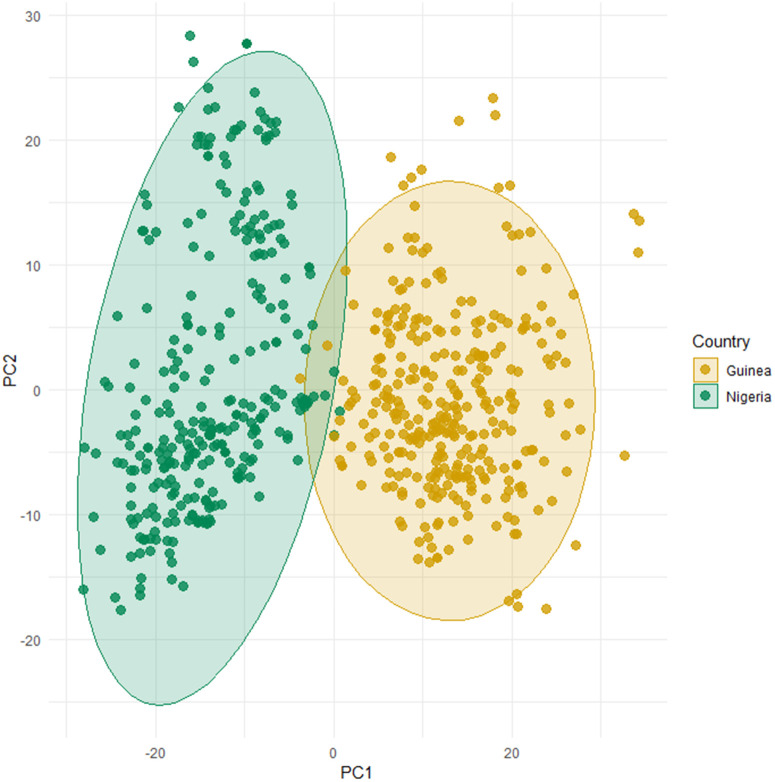
Principal component analysis based on Manhattan distances calculated from the MHC-I repertoire of *M. natalensis* captured in Nigeria (green), where LASV lineage II occurs, and Guinea (yellow), where LASV IV circulates.

Based on physicochemical properties of the 14 PSSs, of which seven aligned perfectly with the human HLA exon 2 PSSs (S1 Appendix), the alleles clustered into 19 MHC-I supertypes with each individual featuring approximately 14 supertypes (± 2.5 SD; t-test: p-value = 0.464). Alleles grouped in the same supertype likely share functionally similar binding characteristics. MHC-I allelic and supertype composition differed between the countries with distinct LASV lineages (Alleles - R2 = 0.16; p-value <0.001; Supertypes - R2 = 0.05; p-value <0.001; Fig 3) and between sampling locations (Alleles - R2 = 0.26; p-value <0.001; Supertypes - R2 = 0.13, p-value <0.001; S2 Fig). While this is in large part due to different alleles found only in one or the other country, 64 alleles are shared between the countries though at different frequencies (Fig 2). For instance, while the four most frequent MHC-I alleles observed in Nigerian *M. natalensis* were also relatively frequent in Guinea, the fifth and sixth most common alleles, ManaMHC-I*005 and ManaMHC-I*006, were never found in Guinea. Noticeable are several alleles rarely found in Nigeria that count among the more common alleles in Guinea (e.g., ManaMHC-I*204; ManaMHC-I*233; ManaMHC-I*242; ManaMHC-I*275). A total of 119 alleles are unique to Guinea, whereas 210 are unique to Nigeria.

### 3.2. Associations between MHC-I and active or cleared LASV infection

**Fig 4:**
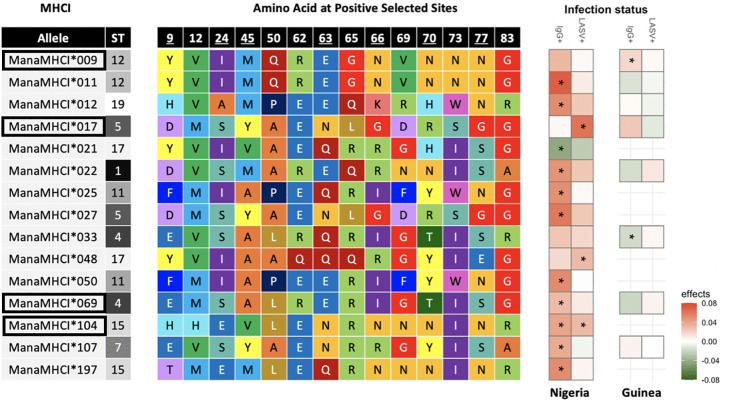
Amino-acid panel of positively selected sites for each MHC-I allele and co-occurrence results between alleles and LASV infection in *Mastomys natalensis* rodents. Supertype assignment is given after the name of each allele. Underlined PSSs indicate sites also under positive selection in the human HLA exon 2. Asterisks indicate statistically significant associations (*p* < 0.01). Positive associations (in red) between specific MHC alleles and LASV-positive rodents, and/or negative associations (in green) between alleles and IgG-positive rodents, suggest susceptibility and active, ongoing infection. In contrast, positive associations with IgG-positive rodents, and/or negative associations with LASV-positive individuals, are indicative of resistance and effective viral clearance. Highlighted alleles (ManaMHC-I*009, *017, *069, and *104) are those for which associations with infection status were also confirmed in our generalized linear mixed-effects models.

The co-occurrence analysis reported three alleles in Nigeria (i.e., ManaMHC-I*017; ManaMHC-I*048 and ManaMHC-I*104) and none in Guinea to be associated with an active LASV infection (Fig 4; S1 Table). Yet, one allele in Guinea (i.e., ManaMHC-I*009) and ten alleles in Nigeria (i.e., ManaMHC-I*011; ManaMHC-I*012; ManaMHC-I*022; ManaMHC-I*025; ManaMHC-I*027; ManaMHC-I*050; ManaMHC-I*069; ManaMHC-I*104; ManaMHC-I*107; ManaMHC-I*197) were more frequently associated with a positive LASV-IgG result than by chance (S2 Table). Only one allele in either country (i.e., ManaMHC-I*021 in Nigeria; ManaMHC-I*033 in Guinea) was less likely to be found in IgG-positive *M. natalensis*. It is noteworthy that in contrast to the present results, ManaMHC-I*009 and ManaMHC-I*021 were previously associated with a higher chance to be found in LASV-positive individuals (Olayemi et al., 2024). Aligning with previous results however, ManaMHC-I*012 and ManaMHC-I*104 were also connected to a higher chance of being found in IgG-positive *M. natalensis* *in the present study*. MHC-I supertype 5 and 18 were positively associated with an active LASV infection in Nigeria and Guinea, respectively (S3 Fig, S1 Table). IgG positive individuals were found more often than expected by chance to carry MHC-I supertype 15 and 5 in Nigeria and Guinea, respectively (S3 Fig, S2 Table). Effect sizes for MHC-I supertypes were, however, notably smaller than those for single alleles.

**Fig 5:**
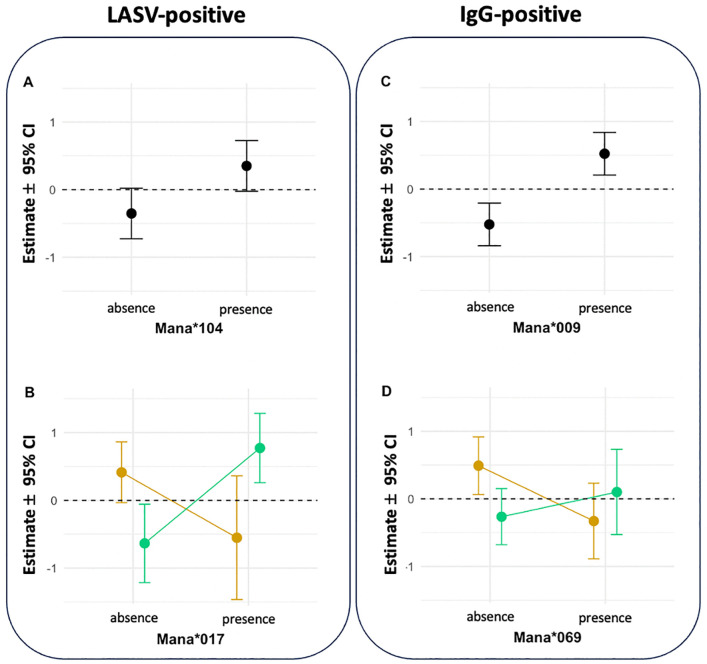
Effects of candidate alleles on probability to be detected positive for an active (LASV-positive) or cleared (IgG-positive) infection calculated from generalised linear mixed effect models. Panels A & C show a positive relationship between the presence of alleles Mana*104, *009 and LASV-positive and IgG-positive animals, respectively. Panels B & D show interactions, resulting in contrasting relationships across countries, between Mana*017, *069 and LASV infection status. Interactions are coloured in green for LASV lineage II found in Nigeria and in yellow for LASV lineage IV found in Guinea.

From these candidate alleles, four alleles had their effect confirmed using a generalised linear mixed effect model that included other biologically relevant covariates (Fig 5; S3 & S4 Tables): Individuals carrying ManaMHC-I*104 had a marginally higher chance of being LASV+ in Nigeria, where the allele occurs (Estimate: 0.99 ± 0.42 SE; p-value = 0.058; Fig 5A). The allele ManaMHC-I*17 was found in both countries but carrying the allele meant a higher risk of being LASV positive in Nigeria and a slightly lower risk in Guinea (interaction - Estimate: 2.37 ± 0.74 SE; p-value = 0.013; Fig 5B). Approximately 16% of *M. natalensis* carry ManaMHC-I*17 in Guinea compared to 33% in Nigeria. A higher chance of detecting IgG antibodies was associated with carrying ManaMHC-I*009 (Estimate: 1.63 ± 0.55 SE; p-value = 0.007; Fig 5C), which is found in both countries. Individuals carrying ManaMHC-I*069 had a lower chance of being IgG positive in Guinea, while the chances were similar whether or not an individual carried ManaMHC-I*069 in Nigeria (interaction - Estimate: 1.18 ± 0.51 SE; p-value = 0.036; Fig 5D).

Neither candidate MHC-I supertype from the co-occurrence analysis was associated with an active LASV infection in the generalised linear mixed effect models (S5 Table). MHC-I supertype 5 and 15 were found more commonly in IgG positive *M. natalensis* (S6 Table). Across all models sex rarely and allelic/supertype diversity never predicted active or past infections (S3-S6 Tables). Higher eye lens weight was associated with an increased likelihood of being IgG positive (S4, S6 Tables).

## 4. Discussion

*Mastomys natalensis* is the primary reservoir for LASV causing viral haemorrhagic fevers in humans. While the virus’s diversification into regional lineages is well understood [6], the consequences for local adaptation of the reservoir host remained unknown. We examined the allelic and functional diversity of the highly polymorphic MHC class I region of the Major Histocompatibility Complex in *M. natalensis* captured in Nigeria and Guinea, where LASV lineage II and IV occur, respectively. Even though much of the allelic and functional MHC-I diversity was shared between countries, the frequencies varied and individual MHC profiles differed markedly. Furthermore, some MHC-LASV associations were shared seemingly irrespective of LASV lineage (e.g., ManaMHC-I*104; ManaMHC-I*009), while others showed divergent associations (e.g., ManaMHC-I*017, ManaMHC-I*069). Collectively, our findings suggest local adaptation to regional LASV lineages in *M. natalensis*.

The MHC-I diversity of *M. natalensis* reached 393 alleles identified across both populations in a total of 665 animals. Such a high number was expected since our previous investigation of 189 *M. natalensis* from Nigeria identified 193 MHC-I alleles alone [37]. Such exceptional MHC-I diversity was also found in other rodent species including *Mus musculus* [72], *Nannomys setulosus* [73] and the closely related *Mastomys erythroleucus* [37]. Our current estimate increases the number of MHC-I loci in *Mastomys natalensis* from 22 to at least 26, while reducing the number of functional allele clusters (i.e., supertypes) from 24 to 19 [37]. The increase in estimated loci is likely due to improved accuracy resulting from our substantially larger sample size. Small sample sizes inherently limit insights in wildlife species with highly diverse MHC genetics [38,74,75]. Similarly, the reduction in MHC-I supertypes likely arose from using a bootstrapped clustering algorithm [62], which improved manual approaches used in the past. Hence, our MHC-I diversity estimates for *M. natalensis* embody a refinement of previous attempts without setting a definite ceiling to its diversity throughout the species’ range.

In addition, and similar to other widely distributed animals [72,76], the vast range of *M. natalensis* in Sub-Saharan Africa helps explain its high MHC-I diversity. As predicted by theory [14,77], the pool of MHC alleles is much larger in the metapopulation than in each subpopulation, because each species is exposed to a larger number of pathogens across its entire range than within any single subregion. Indeed, of the 393 alleles identified, 119 were unique to Guinea whereas 210 were only found in Nigeria. Besides, frequencies of shared alleles varied: alleles common in Nigeria were rare in Guinea, and vice versa. Some of the differences in MHC-I allele frequencies and individual MHC-I profiles between Guinea and Nigeria can be statistically explained by the distinct LASV lineages circulating in each country - lineage IV is prevalent in Guinea, whereas lineage II dominates in Nigeria [39,43]. Moreover, the genetic diversity of the LASV lineages appears linked to the adaptive potential of *M. natalensis* in each region.

Pathogens beyond arenaviruses are scarcely looked for in *M. natalensis* even though several herpesviruses and one novel polyomavirus were reported from countries neighbouring Guinea [78]. In Nigeria, picornaviruses and hepaciviruses are also found co-infecting LASV-positive *M. natalensis* (S7 Table). Consequently, the divergence in MHC-I pools likely betrays broader differences in the overall pathogen communities between the two regions. This pattern is indicative of local adaptation and repeatedly found among cosmopolitan host species [79–82]. Still, stronger evidence for local adaptation are divergent resistance and fitness effects of alleles [22]. Consistent with this, individuals carrying ManaMHC-I*017 were less likely to be LASV positive in Guinea, but more likely in Nigeria. Fewer than a fifth of all individuals carried the seemingly beneficial ManaMHC-I*017 in Guinea, while the allele was found in a third of individuals in Nigeria. However, given the diversity of MHC-I alleles, the limited associations with active or past LASV infection suggest that LASV-mediated selection on MHC-I may be relatively weak. One explanation lies within the transmission ecology of LASV. If LASV antigens are encountered during thymic T-cell development (i.e., in the case of vertical transmission between mother and pup), LASV-derived peptides may be treated as self-like, leading to deletion of high-affinity LASV-specific CD8 + T-cells and thereby reducing LASV-mediated selection on immune genes [83,84]. This hypothesis underscores the need to test whether LASV-mediated selection on *M. natalensis* MHC exceeds expectations under neutral evolution [16].

We think our work is timely because it showcases the importance of funding multi-year ecological and epidemiological surveillance - a cornerstone for tracking wildlife diseases with zoonotic potential [85–87]. Additionally, we illustrate that greater insight into the eco-immunological mechanisms may be gleaned from concurrent (immuno-)genetic monitoring [88–90]. While we fall short of including neutrally evolving markers to distinguish between mechanisms of pathogen-mediated balancing selection and have to rely on uneven sampling throughout the multi-year study, our work is the most complete catalogue of adaptive allelic and functional diversity in the main rodent reservoir of Lassa virus to date and is able to link MHC alleles/supertypes to resistance and fitness effects. In sum, our work provides evidence that regional LASV lineages contribute to the divergence in MHC-I repertoire and local adaptation processes in *M. natalensis*’ West-African range.

## Supporting information

S1 Appendix Genetic sequences and frequencies of MHC-I alleles. (XLSX)

S1 Fig MHCI gene tree. (PDF)

S2 Fig Non-metric multidimensional scaling plot based on Manhattan distances calculated from the MHC-I repertoire from *M. natalensis.* (PDF)

S3 Fig Co-occurrence results as heatmap. (PDF)

S1 Table Co-occurrence results for associating common MHC-I alleles with active LASV infections in Nigerian and Guinean *M. natalensis.* (PDF)

S2 Table Co-occurrence results for associating common MHC-I alleles with active LASV infections in Nigerian and Guinean *M. natalensis.* (PDF)

S3 Table Generalised linear mixed effect model results for the effect of MHC genetics, host sex, country and eye lens weight on LASV detection. (PDF)

S4 Table Generalised linear mixed effect model results for the effect of MHC genetics, host sex, country and eye lens weight on IgG detection. (PDF)

S5 Table Generalised linear mixed effect model results for the effect of MHC supertypes, host sex, country and eye lens weight on LASV detection. (PDF)

S6 Table Generalised linear mixed effect model results for the effect of MHC supertypes, host sex, country and eye lens weight on IgG detection. (PDF)

S7 Table Other RNA viruses detected in LASV-positive *Mastomys natalensis* individuals in localities endemic for LASV lineage II within Nigeria. (PDF)
