# Supplementary figures and images for: Regional Lassa virus lineages select for divergent MHC-I repertoires in Mastomys natalensis rodents
Source: PLoS Pathog. 2026 Apr 17;22(4):e1014121. doi: 10.1371/journal.ppat.1014121 (PMC13124061; doi:10.1371/journal.ppat.1014121)

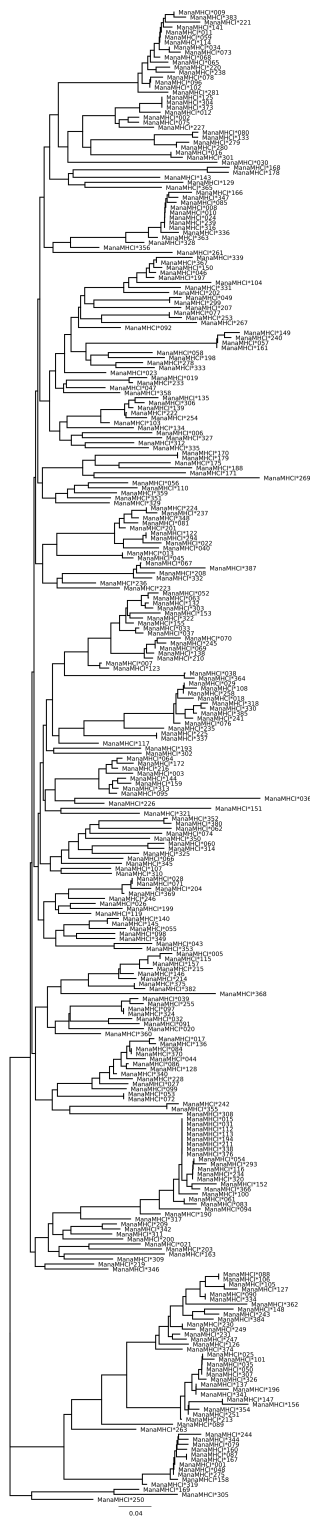

**S1 Fig.** Gene tree constructed from all amino acid alleles of *M. natalensis*.

Supplement: S1 Fig — (PDF) [file ppat.1014121.s002.pdf]
