# Supplementary material for: Regional Lassa virus lineages select for divergent MHC-I repertoires in Mastomys natalensis rodents
Source: PLoS Pathog. 2026 Apr 17;22(4):e1014121. doi: 10.1371/journal.ppat.1014121 (PMC13124061; doi:10.1371/journal.ppat.1014121)
Supplement: S2 Fig — (PDF) [file ppat.1014121.s003.pdf]

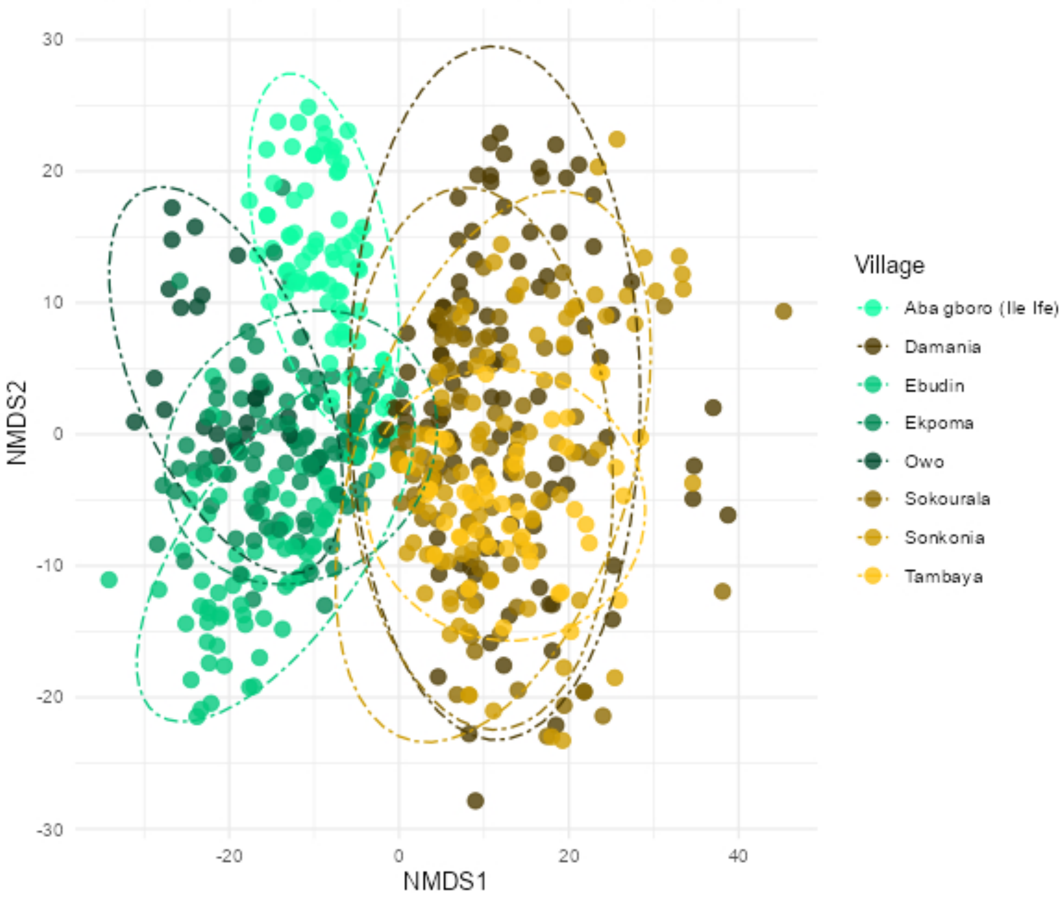

S2 Fig. Non-metric multidimensional scaling plot based on Manhattan distances calculated from the MHC-I repertoire from *M. natalensis* captured in four locations in Guinea (yellow palette), where LASV lineage II occurs, and four locations in Nigeria (green palette), where LASV IV is most prevalent.
