# Supplementary material for: Regional Lassa virus lineages select for divergent MHC-I repertoires in Mastomys natalensis rodents
Source: PLoS Pathog. 2026 Apr 17;22(4):e1014121. doi: 10.1371/journal.ppat.1014121 (PMC13124061; doi:10.1371/journal.ppat.1014121)
Supplement: S3 Fig — (PDF) [file ppat.1014121.s004.pdf]

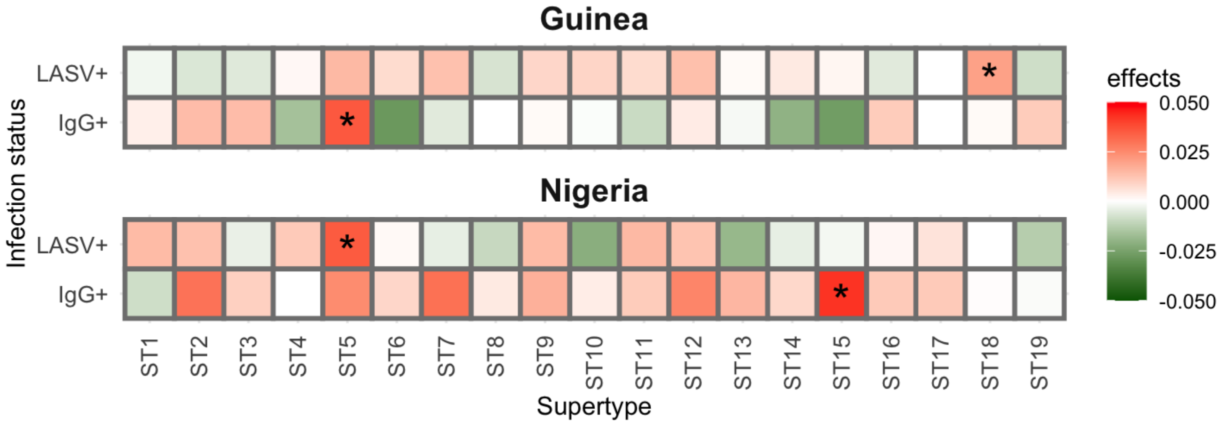


**S3 Fig.** Co-occurrence results as heatmap in which a higher likelihood of finding a specific MHC-I supertypes with an active LASV infection or antibodies from a previous infection indicates susceptibility (red), while a lower likelihood of co-occurrence suggests resistance (green). Results conservatively show p-values <0.01.
