# Supplementary material for: Regional Lassa virus lineages select for divergent MHC-I repertoires in Mastomys natalensis rodents
Source: PLoS Pathog. 2026 Apr 17;22(4):e1014121. doi: 10.1371/journal.ppat.1014121 (PMC13124061; doi:10.1371/journal.ppat.1014121)
Supplement: S1 Table — (PDF) [file ppat.1014121.s005.pdf]

S1 Table. Co-occurrence results for associating common MHC-I alleles with active LASV infections in Nigerian and Guinean *M. natalensis*.

**a) Co-occurrence results for frequent alleles in Nigeria**					
**prob_cooccur**	**p_lt**	**p_gt**	**sp1_name**	**sp2_name**	**effects**
0.1740	0.8910	0.2996	LASV_positive	ManaMHC_001	0.0063
0.1490	0.1377	0.9317	LASV_positive	ManaMHC_002	-0.0140
0.0940	0.1282	0.9306	LASV_positive	ManaMHC_003	-0.0171
0.0940	0.8216	0.2814	LASV_positive	ManaMHC_004	0.0099
0.0980	0.6000	0.5356	LASV_positive	ManaMHC_005	0.0009
0.0880	0.3683	0.7520	LASV_positive	ManaMHC_006	-0.0068
0.0440	0.1727	0.9153	LASV_positive	ManaMHC_007	-0.0126
0.0760	0.9476	0.1008	LASV_positive	ManaMHC_008	0.0189
0.0730	0.6696	0.4646	LASV_positive	ManaMHC_009	0.0032
0.0540	0.4435	0.7006	LASV_positive	ManaMHC_010	-0.0041
0.0770	0.8064	0.3020	LASV_positive	ManaMHC_011	0.0090
0.0300	0.9955	0.0142	LASV_positive	ManaMHC_012	0.0243
0.0390	0.9423	0.1200	LASV_positive	ManaMHC_013	0.0149
0.0630	0.8188	0.2899	LASV_positive	ManaMHC_014	0.0090
0.0350	0.2970	0.8406	LASV_positive	ManaMHC_015	-0.0081
0.0480	0.2063	0.8913	LASV_positive	ManaMHC_016	-0.0117
**0.0690**	**100000.0000**	**0.0000**	**LASV_positive**	**ManaMHC_017**	**0.0617**
0.0690	0.7818	0.3349	LASV_positive	ManaMHC_018	0.0077
0.0740	0.8569	0.2359	LASV_positive	ManaMHC_019	0.0113
0.0720	0.8220	0.2831	LASV_positive	ManaMHC_020	0.0095
0.0700	0.0353	0.9856	LASV_positive	ManaMHC_021	-0.0248
0.0650	0.9853	0.0337	LASV_positive	ManaMHC_022	0.0252
0.0560	0.2306	0.8696	LASV_positive	ManaMHC_023	-0.0113
0.0490	0.4344	0.7124	LASV_positive	ManaMHC_024	-0.0045
0.0370	0.7002	0.4592	LASV_positive	ManaMHC_025	0.0032
0.0660	0.3542	0.7699	LASV_positive	ManaMHC_026	-0.0072
0.0560	0.9864	0.0322	LASV_positive	ManaMHC_027	0.0248
0.0200	0.5519	0.6697	LASV_positive	ManaMHC_028	-0.0018
0.0840	0.9901	0.0235	LASV_positive	ManaMHC_029	0.0284
0.0180	0.8175	0.3621	LASV_positive	ManaMHC_030	0.0045
0.0200	0.8839	0.2506	LASV_positive	ManaMHC_031	0.0072
0.0200	0.5519	0.6697	LASV_positive	ManaMHC_032	-0.0018
0.0320	0.9769	0.0581	LASV_positive	ManaMHC_033	0.0180

0.0200	0.0429	0.9940	LASV_positive	ManaMHC_034	-0.0153
0.0280	0.9918	0.0245	LASV_positive	ManaMHC_035	0.0216
0.0480	0.2063	0.8913	LASV_positive	ManaMHC_036	-0.0117
0.0300	0.9613	0.0913	LASV_positive	ManaMHC_046	0.0153
**0.0500**	**0.9971**	**0.0084**	**LASV_positive**	**ManaMHC_048**	**0.0306**
0.0610	0.1401	0.9284	LASV_positive	ManaMHC_049	-0.0153
0.0460	0.5535	0.6047	LASV_positive	ManaMHC_050	-0.0009
0.0420	0.3838	0.7640	LASV_positive	ManaMHC_053	-0.0059
0.0170	0.2285	0.9243	LASV_positive	ManaMHC_054	-0.0081
0.0510	0.5302	0.6222	LASV_positive	ManaMHC_056	-0.0018
0.0620	0.9917	0.0205	LASV_positive	ManaMHC_057	0.0279
0.0120	0.4852	0.7829	LASV_positive	ManaMHC_059	-0.0027
0.0170	0.6807	0.5466	LASV_positive	ManaMHC_063	0.0009
0.0420	0.3838	0.7640	LASV_positive	ManaMHC_067	-0.0059
0.0260	0.9004	0.2057	LASV_positive	ManaMHC_069	0.0095
0.0500	0.4058	0.7365	LASV_positive	ManaMHC_071	-0.0054
0.0090	0.6541	0.6514	LASV_positive	ManaMHC_081	-0.0005
0.0180	0.6379	0.5901	LASV_positive	ManaMHC_084	0.0000
0.0130	0.4337	0.8163	LASV_positive	ManaMHC_087	-0.0036
0.0030	0.9075	0.4687	LASV_positive	ManaMHC_090	0.0018
0.0030	0.5314	100000	LASV_positive	ManaMHC_094	-0.0027
0.0220	0.0237	0.9971	LASV_positive	ManaMHC_100	-0.0176
**0.0550**	**0.9967**	**0.0091**	**LASV_positive**	**ManaMHC_104**	**0.0311**
0.0320	0.3883	0.7747	LASV_positive	ManaMHC_107	-0.0054
0.0240	0.0158	0.9982	LASV_positive	ManaMHC_108	-0.0194
0.0160	0.0910	0.9847	LASV_positive	ManaMHC_122	-0.0117
0.0140	0.6429	0.6146	LASV_positive	ManaMHC_125	0.0000
0.0540	0.7295	0.4064	LASV_positive	ManaMHC_197	0.0050
0.0370	0.9945	0.0159	LASV_positive	ManaMHC_198	0.0257
0.0320	0.8532	0.2690	LASV_positive	ManaMHC_201	0.0081
0.0290	0.0750	0.9773	LASV_positive	ManaMHC_202	-0.0153
0.0140	0.3854	0.8451	LASV_positive	ManaMHC_204	-0.0045
0.0110	0.9962	0.0197	LASV_positive	ManaMHC_216	0.0158
0.0120	0.7391	0.5148	LASV_positive	ManaMHC_225	0.0018
0.0090	0.4042	0.8831	LASV_positive	ManaMHC_227	-0.0041
0.0110	0.5396	0.7446	LASV_positive	ManaMHC_230	-0.0023
0.0090	0.6541	0.6514	LASV_positive	ManaMHC_233	-0.0005
0.0070	0.8235	0.4668	LASV_positive	ManaMHC_234	0.0023
0.0010	0.8108	100000	LASV_positive	ManaMHC_242	-0.0009

**b) Co-occurrence results for frequent alleles in Guinea**					
**prob_cooccur**	**p_lt**	**p_gt**	**sp1_name**	**sp2_name**	**effects**
0.0870	0.1612	0.9088	LASV_positive	ManaMHC_001	-0.0123
0.0770	0.7175	0.4082	LASV_positive	ManaMHC_002	0.0045
0.0560	0.2983	0.8129	LASV_positive	ManaMHC_003	-0.0074
0.0950	0.5057	0.6331	LASV_positive	ManaMHC_004	-0.0015
0.0160	0.9541	0.1163	LASV_positive	ManaMHC_007	0.0100
0.0440	0.4703	0.6751	LASV_positive	ManaMHC_008	-0.0026
0.0080	0.8321	0.3907	LASV_positive	ManaMHC_009	0.0030
0.0480	0.8266	0.2802	LASV_positive	ManaMHC_010	0.0078
0.0300	0.3975	0.7572	LASV_positive	ManaMHC_011	-0.0045
0.0430	0.3452	0.7837	LASV_positive	ManaMHC_012	-0.0059
0.0530	0.8750	0.2119	LASV_positive	ManaMHC_013	0.0100
0.0320	0.4906	0.6720	LASV_positive	ManaMHC_014	-0.0022
0.0220	0.1150	0.9611	LASV_positive	ManaMHC_017	-0.0108
0.0410	0.2681	0.8448	LASV_positive	ManaMHC_018	-0.0082
0.0900	0.8847	0.2001	LASV_positive	ManaMHC_022	0.0108
0.0640	0.9844	0.0351	LASV_positive	ManaMHC_024	0.0216
0.0180	0.4649	0.7378	LASV_positive	ManaMHC_030	-0.0030
0.0320	0.6512	0.5094	LASV_positive	ManaMHC_032	0.0015
0.0110	0.8421	0.3442	LASV_positive	ManaMHC_033	0.0037
0.0220	0.1285	0.9554	LASV_positive	ManaMHC_034	-0.0104
0.0100	0.8902	0.2703	LASV_positive	ManaMHC_035	0.0048
0.0030	0.8390	0.5558	LASV_positive	ManaMHC_036	0.0011
0.0200	0.3478	0.8231	LASV_positive	ManaMHC_038	-0.0052
0.0390	0.1263	0.9412	LASV_positive	ManaMHC_049	-0.0126
0.0310	0.1133	0.9534	LASV_positive	ManaMHC_053	-0.0123
0.0860	0.5597	0.5777	LASV_positive	ManaMHC_054	0.0000
0.0400	0.6055	0.5461	LASV_positive	ManaMHC_059	0.0004
0.0680	0.3943	0.7300	LASV_positive	ManaMHC_063	-0.0048
0.0350	0.7902	0.3387	LASV_positive	ManaMHC_069	0.0056
0.0320	0.4906	0.6720	LASV_positive	ManaMHC_081	-0.0022
0.0400	0.8460	0.2592	LASV_positive	ManaMHC_084	0.0078
0.0570	0.3705	0.7536	LASV_positive	ManaMHC_087	-0.0056
0.0300	0.4220	0.7375	LASV_positive	ManaMHC_090	-0.0037
0.0390	0.3689	0.7688	LASV_positive	ManaMHC_094	-0.0052
0.0270	0.9194	0.1622	LASV_positive	ManaMHC_100	0.0100

0.0180	0.6373	0.5659	LASV_positive	ManaMHC_107	0.0004
0.0470	0.7561	0.3703	LASV_positive	ManaMHC_108	0.0052
0.0280	0.9082	0.1798	LASV_positive	ManaMHC_122	0.0097
0.0360	0.3277	0.8052	LASV_positive	ManaMHC_125	-0.0063
0.0510	0.6210	0.5205	LASV_positive	ManaMHC_201	0.0011
0.0450	0.9496	0.1000	LASV_positive	ManaMHC_202	0.0149
0.0600	0.8850	0.1963	LASV_positive	ManaMHC_204	0.0108
0.0480	0.4641	0.6774	LASV_positive	ManaMHC_216	-0.0030
0.0310	0.3736	0.7759	LASV_positive	ManaMHC_225	-0.0048
0.0750	0.7970	0.3128	LASV_positive	ManaMHC_227	0.0071
0.0500	0.9231	0.1415	LASV_positive	ManaMHC_230	0.0130
0.1060	0.7672	0.3660	LASV_positive	ManaMHC_233	0.0052
0.0210	0.2963	0.8568	LASV_positive	ManaMHC_234	-0.0063
0.0710	0.4082	0.7175	LASV_positive	ManaMHC_242	-0.0045
0.0870	0.9271	0.1361	LASV_positive	ManaMHC_275	0.0138
0.0710	0.9308	0.1276	LASV_positive	ManaMHC_299	0.0141
0.0570	0.1482	0.9202	LASV_positive	ManaMHC_300	-0.0130
0.0510	0.4581	0.6801	LASV_positive	ManaMHC_301	-0.0030
0.0540	0.0786	0.9633	LASV_positive	ManaMHC_302	-0.0164
0.0390	0.9738	0.0581	LASV_positive	ManaMHC_303	0.0171
0.0380	0.2675	0.8489	LASV_positive	ManaMHC_304	-0.0078
0.0460	0.3627	0.7666	LASV_positive	ManaMHC_305	-0.0056
0.0450	0.5521	0.5954	LASV_positive	ManaMHC_306	-0.0007
0.0330	0.8553	0.2531	LASV_positive	ManaMHC_308	0.0078
0.0360	0.1948	0.9008	LASV_positive	ManaMHC_309	-0.0100
0.0340	0.5556	0.6056	LASV_positive	ManaMHC_310	-0.0007
0.0380	0.6926	0.4537	LASV_positive	ManaMHC_312	0.0026
0.0380	0.5696	0.5865	LASV_positive	ManaMHC_313	-0.0004
0.0340	0.8244	0.2948	LASV_positive	ManaMHC_314	0.0067
**c) Co-occurrence results for frequent supertypes in Nigeria**					
**prob_cooccur**	**p_lt**	**p_gt**	**sp1_name**	**sp2_name**	**effects**
0.1830	100000.0000	0.2252	LASV_positive	Supertype_17	0.0059
0.1590	0.2125	0.8919	LASV_positive	Supertype_8	-0.0099
0.1210	0.4442	0.6896	LASV_positive	Supertype_14	-0.0041
0.1300	0.4567	0.6823	LASV_positive	Supertype_3	-0.0036
0.1740	0.7004	0.5459	LASV_positive	Supertype_16	0.0018
0.1360	0.9056	0.1797	LASV_positive	Supertype_2	0.0131

0.1440	0.5754	0.5836	LASV_positive	Supertype_18	0.0000
0.1540	0.9305	0.1594	LASV_positive	Supertype_12	0.0126
0.1210	0.1985	0.8844	LASV_positive	Supertype_19	-0.0131
0.1750	100000.0000	0.0243	LASV_positive	Supertype_1	0.0144
0.1570	0.9606	0.1068	LASV_positive	Supertype_9	0.0144
**0.1410**	**0.9998**	**0.0010**	**LASV_positive**	**Supertype_5**	**0.0351**
0.1520	0.6291	0.5408	LASV_positive	Supertype_6	0.0014
0.1340	0.9267	0.1456	LASV_positive	Supertype_11	0.0149
0.1370	0.8800	0.2190	LASV_positive	Supertype_4	0.0113
0.0820	0.1019	0.9478	LASV_positive	Supertype_13	-0.0189
0.1210	0.4442	0.6896	LASV_positive	Supertype_7	-0.0041
0.1120	0.0685	0.9665	LASV_positive	Supertype_10	-0.0216
0.1100	0.5107	0.6253	LASV_positive	Supertype_15	-0.0018
**d) Co-occurrence results for frequent supertypes in Guinea**					
**prob_cooccur**	**p_lt**	**p_gt**	**sp1_name**	**sp2_name**	**effects**
0.1490	100000.0000	100000.0000	LASV_positive	Supertype_17	0.0000
0.1370	0.1848	0.9283	LASV_positive	Supertype_8	-0.0071
0.1000	0.7330	0.4000	LASV_positive	Supertype_14	0.0045
0.0870	0.3624	0.7572	LASV_positive	Supertype_3	-0.0056
0.1320	0.3034	0.8454	LASV_positive	Supertype_16	-0.0052
0.1070	0.3188	0.8003	LASV_positive	Supertype_2	-0.0063
**0.1250**	**0.9995**	**0.0052**	**LASV_positive**	**Supertype_18**	**0.0201**
0.0950	0.9287	0.1364	LASV_positive	Supertype_12	0.0134
0.1280	0.1792	0.9151	LASV_positive	Supertype_19	-0.0086
0.1470	0.3843	0.9412	LASV_positive	Supertype_1	-0.0022
0.1330	0.9523	0.1578	LASV_positive	Supertype_9	0.0086
0.0860	0.9402	0.1150	LASV_positive	Supertype_5	0.0149
0.0710	0.8014	0.3071	LASV_positive	Supertype_6	0.0074
0.1190	0.8620	0.2625	LASV_positive	Supertype_11	0.0074
0.1400	0.7201	0.5653	LASV_positive	Supertype_4	0.0015
0.0030	0.8390	0.5558	LASV_positive	Supertype_13	0.0011
0.1240	0.9779	0.0722	LASV_positive	Supertype_7	0.0130
0.0990	0.8529	0.2497	LASV_positive	Supertype_10	0.0089
0.0870	0.6343	0.5021	LASV_positive	Supertype_15	0.0019
