# Supplementary material for: Regional Lassa virus lineages select for divergent MHC-I repertoires in Mastomys natalensis rodents
Source: PLoS Pathog. 2026 Apr 17;22(4):e1014121. doi: 10.1371/journal.ppat.1014121 (PMC13124061; doi:10.1371/journal.ppat.1014121)
Supplement: S2 Table — (PDF) [file ppat.1014121.s006.pdf]

S2 Table. Co-occurrence results for associating common MHC-I alleles with previous LASV infections in Nigerian and Guinean *M. natalensis*.

**a) Co-occurrence results for frequent alleles in Nigeria**					
**prob_cooccur**	**p_lt**	**p_gt**	**sp1_name**	**sp2_name**	**effects**
0.3310	0.9418	0.1543	IgG_positive	ManaMHC_001	0.0113
0.2840	0.5558	0.5798	IgG_positive	ManaMHC_002	-0.0005
0.1790	0.7880	0.3015	IgG_positive	ManaMHC_003	0.0108
0.1790	0.9495	0.0870	IgG_positive	ManaMHC_004	0.0243
0.1870	0.8720	0.1962	IgG_positive	ManaMHC_005	0.0162
0.1670	0.0976	0.9426	IgG_positive	ManaMHC_006	-0.0230
0.0840	0.7212	0.3979	IgG_positive	ManaMHC_007	0.0059
0.1440	0.7561	0.3412	IgG_positive	ManaMHC_008	0.0086
0.1400	0.9842	0.0312	IgG_positive	ManaMHC_009	0.0315
0.1020	0.5998	0.5225	IgG_positive	ManaMHC_010	0.0014
**0.1460**	**100000.0000**	**0.0000**	**IgG_positive**	**ManaMHC_011**	**0.0703**
**0.0570**	**100000.0000**	**0.0000**	**IgG_positive**	**ManaMHC_012**	**0.0514**
0.0750	0.9110	0.1567	IgG_positive	ManaMHC_013	0.0153
0.1200	0.1078	0.9382	IgG_positive	ManaMHC_014	-0.0212
0.0670	0.0100	0.9967	IgG_positive	ManaMHC_015	-0.0306
0.0910	0.0144	0.9943	IgG_positive	ManaMHC_016	-0.0324
0.1310	0.6494	0.4626	IgG_positive	ManaMHC_017	0.0036
0.1310	0.9828	0.0339	IgG_positive	ManaMHC_018	0.0306
0.1410	0.9605	0.0705	IgG_positive	ManaMHC_019	0.0252
0.1360	0.9338	0.1116	IgG_positive	ManaMHC_020	0.0212
**0.1330**	**0.0040**	**0.9984**	**IgG_positive**	**ManaMHC_021**	**-0.0428**
0.1230	0.9994	0.0015	IgG_positive	ManaMHC_022	0.0477
0.1070	0.0260	0.9881	IgG_positive	ManaMHC_023	-0.0306
0.0940	0.2234	0.8607	IgG_positive	ManaMHC_024	-0.0131
**0.0710**	**1.0000**	**0.0001**	**IgG_positive**	**ManaMHC_025**	**0.0500**
0.1250	0.4727	0.6415	IgG_positive	ManaMHC_026	-0.0032
**0.1070**	**1.0000**	**0.0001**	**IgG_positive**	**ManaMHC_027**	**0.0595**
0.0370	0.2080	0.9021	IgG_positive	ManaMHC_028	-0.0104
0.1610	0.9125	0.1411	IgG_positive	ManaMHC_029	0.0194
0.0340	0.3104	0.8382	IgG_positive	ManaMHC_030	-0.0072
0.0370	0.7145	0.4547	IgG_positive	ManaMHC_031	0.0032
0.0370	0.9715	0.0722	IgG_positive	ManaMHC_032	0.0167
0.0600	0.9887	0.0277	IgG_positive	ManaMHC_033	0.0257

0.0370	0.5454	0.6351	IgG_positive	ManaMHC_034	-0.0014
0.0540	0.0892	0.9609	IgG_positive	ManaMHC_035	-0.0176
0.0910	0.6666	0.4561	IgG_positive	ManaMHC_036	0.0036
0.0570	0.9870	0.0319	IgG_positive	ManaMHC_046	0.0243
0.0960	0.9094	0.1528	IgG_positive	ManaMHC_048	0.0167
0.1150	0.6097	0.5082	IgG_positive	ManaMHC_049	0.0018
**0.0880**	**1.0000**	**0.0002**	**IgG_positive**	**ManaMHC_050**	**0.0518**
0.0800	0.7343	0.3852	IgG_positive	ManaMHC_053	0.0059
0.0320	0.9943	0.0197	IgG_positive	ManaMHC_054	0.0216
0.0970	0.8179	0.2758	IgG_positive	ManaMHC_056	0.0108
0.1180	0.9923	0.0166	IgG_positive	ManaMHC_057	0.0347
0.0230	0.9188	0.1996	IgG_positive	ManaMHC_059	0.0090
0.0320	0.7394	0.4360	IgG_positive	ManaMHC_063	0.0036
0.0800	0.3511	0.7650	IgG_positive	ManaMHC_067	-0.0077
0.0500	0.9981	0.0061	IgG_positive	ManaMHC_069	0.0306
0.0960	0.1163	0.9355	IgG_positive	ManaMHC_071	-0.0194
0.0180	0.6434	0.6066	IgG_positive	ManaMHC_081	0.0000
0.0340	0.3104	0.8382	IgG_positive	ManaMHC_084	-0.0072
0.0240	0.9552	0.1228	IgG_positive	ManaMHC_087	0.0117
0.0050	0.7049	0.7403	IgG_positive	ManaMHC_090	-0.0005
0.0050	100000.0000	0.0457	IgG_positive	ManaMHC_094	0.0086
0.0420	0.6923	0.4712	IgG_positive	ManaMHC_100	0.0027
**0.1040**	**0.9980**	**0.0049**	**IgG_positive**	**ManaMHC_104**	**0.0401**
**0.0620**	**0.9993**	**0.0022**	**IgG_positive**	**ManaMHC_107**	**0.0374**
0.0450	0.8449	0.2735	IgG_positive	ManaMHC_108	0.0086
0.0310	0.7972	0.3659	IgG_positive	ManaMHC_122	0.0054
0.0260	0.4518	0.7486	IgG_positive	ManaMHC_125	-0.0036
**0.1020**	**0.9999**	**0.0005**	**IgG_positive**	**ManaMHC_197**	**0.0509**
0.0710	0.9893	0.0251	IgG_positive	ManaMHC_198	0.0275
0.0620	0.2091	0.8832	IgG_positive	ManaMHC_201	-0.0122
0.0550	0.6891	0.4568	IgG_positive	ManaMHC_202	0.0032
0.0260	0.9939	0.0237	IgG_positive	ManaMHC_204	0.0189
0.0210	0.2453	0.9075	IgG_positive	ManaMHC_216	-0.0077
0.0230	0.3861	0.8110	IgG_positive	ManaMHC_225	-0.0045
0.0160	0.7324	0.5157	IgG_positive	ManaMHC_227	0.0018
0.0210	0.4665	0.7547	IgG_positive	ManaMHC_230	-0.0032
0.0180	0.1741	0.9514	IgG_positive	ManaMHC_233	-0.0090
0.0130	0.6864	0.5995	IgG_positive	ManaMHC_234	0.0005
0.0020	0.6396	100000.0000	IgG_positive	ManaMHC_242	-0.0018

**b) Co-occurrence results for frequent alleles in Guinea**					
**prob_cooccur**	**p_lt**	**p_gt**	**sp1_name**	**sp2_name**	**effects**
0.1670	0.8943	0.1651	IgG_positive	ManaMHC_001	0.0152
0.1490	0.4381	0.6648	IgG_positive	ManaMHC_002	-0.0041
0.1070	0.5671	0.5436	IgG_positive	ManaMHC_003	0.0004
0.1820	0.7619	0.3335	IgG_positive	ManaMHC_004	0.0078
0.0310	0.8312	0.2951	IgG_positive	ManaMHC_007	0.0063
0.0840	0.4901	0.6261	IgG_positive	ManaMHC_008	-0.0022
**0.0160**	**0.9981**	**0.0091**	**IgG_positive**	**ManaMHC_009**	**0.0175**
0.0930	0.9704	0.0543	IgG_positive	ManaMHC_010	0.0227
0.0590	0.1383	0.9246	IgG_positive	ManaMHC_011	-0.0138
0.0830	0.6392	0.4760	IgG_positive	ManaMHC_012	0.0026
0.1020	0.0449	0.9765	IgG_positive	ManaMHC_013	-0.0242
0.0620	0.8294	0.2643	IgG_positive	ManaMHC_014	0.0089
0.0430	0.9952	0.0127	IgG_positive	ManaMHC_017	0.0245
0.0800	0.3889	0.7209	IgG_positive	ManaMHC_018	-0.0056
0.1720	0.1431	0.9099	IgG_positive	ManaMHC_022	-0.0164
0.1230	0.3221	0.7689	IgG_positive	ManaMHC_024	-0.0082
0.0340	0.9155	0.1643	IgG_positive	ManaMHC_030	0.0104
0.0620	0.3634	0.7524	IgG_positive	ManaMHC_032	-0.0059
**0.0210**	**0.0086**	**0.9991**	**IgG_positive**	**ManaMHC_033**	**-0.0175**
0.0420	0.2661	0.8465	IgG_positive	ManaMHC_034	-0.0082
0.0190	0.5870	0.6255	IgG_positive	ManaMHC_035	-0.0007
0.0050	0.5561	0.8175	IgG_positive	ManaMHC_036	-0.0015
0.0380	0.8092	0.3124	IgG_positive	ManaMHC_038	0.0063
0.0740	0.6765	0.4394	IgG_positive	ManaMHC_049	0.0037
0.0600	0.6910	0.4326	IgG_positive	ManaMHC_053	0.0037
0.1650	0.8034	0.2811	IgG_positive	ManaMHC_054	0.0097
0.0780	0.4562	0.6609	IgG_positive	ManaMHC_059	-0.0033
0.1310	0.1009	0.9393	IgG_positive	ManaMHC_063	-0.0193
0.0680	0.0610	0.9699	IgG_positive	ManaMHC_069	-0.0197
0.0620	0.6204	0.5080	IgG_positive	ManaMHC_081	0.0015
0.0780	0.6893	0.4236	IgG_positive	ManaMHC_084	0.0041
0.1110	0.8492	0.2242	IgG_positive	ManaMHC_087	0.0119
0.0570	0.2578	0.8403	IgG_positive	ManaMHC_090	-0.0093
0.0740	0.9138	0.1438	IgG_positive	ManaMHC_094	0.0149
0.0520	0.0535	0.9766	IgG_positive	ManaMHC_100	-0.0186

0.0350	0.8026	0.3261	IgG_positive	ManaMHC_107	0.0059
0.0900	0.2997	0.7935	IgG_positive	ManaMHC_108	-0.0086
0.0530	0.0442	0.9812	IgG_positive	ManaMHC_122	-0.0197
0.0690	0.0963	0.9486	IgG_positive	ManaMHC_125	-0.0171
0.0980	0.8163	0.2678	IgG_positive	ManaMHC_201	0.0100
0.0860	0.5401	0.5767	IgG_positive	ManaMHC_202	-0.0007
0.1150	0.0379	0.9802	IgG_positive	ManaMHC_204	-0.0257
0.0920	0.3760	0.7282	IgG_positive	ManaMHC_216	-0.0059
0.0600	0.9635	0.0706	IgG_positive	ManaMHC_225	0.0186
0.1440	0.5913	0.5154	IgG_positive	ManaMHC_227	0.0015
0.0970	0.0295	0.9855	IgG_positive	ManaMHC_230	-0.0260
0.2040	0.3290	0.7697	IgG_positive	ManaMHC_233	-0.0074
0.0400	0.9831	0.0395	IgG_positive	ManaMHC_234	0.0190
0.1370	0.4546	0.6495	IgG_positive	ManaMHC_242	-0.0033
0.1670	0.4508	0.6542	IgG_positive	ManaMHC_275	-0.0033
0.1370	0.0411	0.9778	IgG_positive	ManaMHC_299	-0.0257
0.1110	0.6853	0.4182	IgG_positive	ManaMHC_300	0.0045
0.0990	0.2752	0.8115	IgG_positive	ManaMHC_301	-0.0097
0.1030	0.1148	0.9315	IgG_positive	ManaMHC_302	-0.0178
0.0740	0.8562	0.2232	IgG_positive	ManaMHC_303	0.0112
0.0720	0.8273	0.2617	IgG_positive	ManaMHC_304	0.0093
0.0890	0.3289	0.7694	IgG_positive	ManaMHC_305	-0.0074
0.0870	0.0383	0.9811	IgG_positive	ManaMHC_306	-0.0242
0.0640	0.4184	0.7030	IgG_positive	ManaMHC_308	-0.0045
0.0690	0.2557	0.8360	IgG_positive	ManaMHC_309	-0.0097
0.0660	0.8118	0.2845	IgG_positive	ManaMHC_310	0.0086
0.0730	0.8760	0.1971	IgG_positive	ManaMHC_312	0.0119
0.0720	0.0297	0.9866	IgG_positive	ManaMHC_313	-0.0242
0.0660	0.9344	0.1156	IgG_positive	ManaMHC_314	0.0160
**c) Co-occurrence results for frequent supertypes in Nigeria**					
**prob_cooccur**	**p_lt**	**p_gt**	**sp1_name**	**sp2_name**	**effects**
0.3490	100000.0000	0.0415	IgG_positive	Supertype_17	0.0113
0.3020	0.7087	0.4336	IgG_positive	Supertype_8	0.0045
0.2310	0.7504	0.3507	IgG_positive	Supertype_14	0.0081
0.2470	0.7933	0.3033	IgG_positive	Supertype_3	0.0099
0.3310	0.9418	0.1543	IgG_positive	Supertype_16	0.0113
0.2580	0.9883	0.0258	IgG_positive	Supertype_2	0.0302

0.2740	0.5748	0.5554	IgG_positive	Supertype_18	0.0005
0.2940	0.9900	0.0263	IgG_positive	Supertype_12	0.0261
0.2310	0.5366	0.5789	IgG_positive	Supertype_19	-0.0009
0.3330	0.2324	0.8926	IgG_positive	Supertype_1	-0.0086
0.2990	0.9427	0.1168	IgG_positive	Supertype_9	0.0167
0.2680	0.9750	0.0518	IgG_positive	Supertype_5	0.0248
0.2890	0.7946	0.3202	IgG_positive	Supertype_6	0.0086
0.2550	0.8151	0.2787	IgG_positive	Supertype_11	0.0108
0.2610	0.5574	0.5669	IgG_positive	Supertype_4	0.0000
0.1560	0.8647	0.2060	IgG_positive	Supertype_13	0.0153
0.2310	0.9843	0.0318	IgG_positive	Supertype_7	0.0306
0.2130	0.6425	0.4678	IgG_positive	Supertype_10	0.0036
**0.2090**	**0.9979**	**0.0050**	**IgG_positive**	**Supertype_15**	**0.0428**
**c) cooccurrence results for frequent supertypes in Guinea**					
**prob_cooccur**	**p_lt**	**p_gt**	**sp1_name**	**sp2_name**	**effects**
0.2860	100000.0000	100000.0000	IgG_positive	Supertype_17	0.0000
0.2640	0.5897	0.6079	IgG_positive	Supertype_8	0.0000
0.1920	0.0758	0.9569	IgG_positive	Supertype_14	-0.0204
0.1680	0.8795	0.1851	IgG_positive	Supertype_3	0.0141
0.2530	0.9274	0.1579	IgG_positive	Supertype_16	0.0108
0.2050	0.9000	0.1649	IgG_positive	Supertype_2	0.0141
0.2400	0.6105	0.5352	IgG_positive	Supertype_18	0.0011
0.1820	0.6665	0.4407	IgG_positive	Supertype_12	0.0041
0.2460	0.9075	0.1795	IgG_positive	Supertype_19	0.0108
0.2830	100000.0000	0.3620	IgG_positive	Supertype_1	0.0033
0.2550	0.6276	0.5441	IgG_positive	Supertype_9	0.0011
**0.1650**	**0.9974**	**0.0059**	**IgG_positive**	**Supertype_5**	**0.0357**
0.1360	0.0266	0.9864	IgG_positive	Supertype_6	-0.0283
0.2290	0.2435	0.8472	IgG_positive	Supertype_11	-0.0093
0.2690	0.0159	0.9962	IgG_positive	Supertype_4	-0.0164
0.0050	0.5561	0.8175	IgG_positive	Supertype_13	-0.0015
0.2390	0.3649	0.7591	IgG_positive	Supertype_7	-0.0052
0.1900	0.5272	0.5860	IgG_positive	Supertype_10	-0.0007
0.1680	0.0331	0.9826	IgG_positive	Supertype_15	-0.0268
