# Supplementary material for: Regional Lassa virus lineages select for divergent MHC-I repertoires in Mastomys natalensis rodents
Source: PLoS Pathog. 2026 Apr 17;22(4):e1014121. doi: 10.1371/journal.ppat.1014121 (PMC13124061; doi:10.1371/journal.ppat.1014121)
Supplement: S3 Table — (PDF) [file ppat.1014121.s007.pdf]

S3 Table. Generalised linear mixed effect model results for the effect of MHC genetics, host sex, country and eye lens weight on LASV detection.

**a)**		**Estimate**	**Std. error**	**p-values**	**fdr-corrected**
(Intercept)		-2,121,605	0.712384	0.00290	0.013
ManaMHC_017		-0.965716	0.625175	0.12242	0.233
CountryNigeria		-1,049,571	0.372976	0.00489	**0.019**
Number_alleles		0.008732	0.019360	0.65198	0.851
ELW		0.004736	0.017613	0.78803	0.881
SexM		-0.110335	0.260319	0.67168	0.851
ManaMHC_017:CountryNigeria		2,374,130	0.741536	0.00137	**0.013**
**b)**		**Estimate**	**Std. error**	**p-values**	**fdr-corrected**
(Intercept)		-2,215,317	0.691394	0.00135	0.013
ManaMHC_048		0.762325	0.407425	0.06133	0.166
CountryNigeria		-0.488866	0.309268	0.11394	0.233
Number_alleles		0.009628	0.018636	0.60540	0.851
ELW		0.002633	0.016865	0.87596	0.918
SexM		-0.122951	0.257670	0.63325	0.851
**c)**		**Estimate**	**Std. error**	**p-values**	**fdr-corrected**
(Intercept)		-2,094,003	0.682511	0.00215	0.013
ManaMHC_104		0.989122	0.423411	0.01949	0.058
CountryNigeria		-0.509498	0.305136	0.09497	0.226
Number_alleles		0.006283	0.018377	0.73244	0.870
ELW		0.001756	0.017075	0.91808	0.918
SexM		-0.127902	0.258989	0.62141	0.851
