# Supplementary material for: Regional Lassa virus lineages select for divergent MHC-I repertoires in Mastomys natalensis rodents
Source: PLoS Pathog. 2026 Apr 17;22(4):e1014121. doi: 10.1371/journal.ppat.1014121 (PMC13124061; doi:10.1371/journal.ppat.1014121)
Supplement: S4 Table — (PDF) [file ppat.1014121.s008.pdf]

S4 Table. Generalised linear mixed effect model results for the effect of MHC genetics, host sex, country and eye lens weight on IgG detection.

a)		Estimate	Std. error	p-values	fdr-corrected
(Intercept)		-295,202	0.58512	4.53e-07	**0.00000177**
ManaMHC_009		163,014	0.55276	0.00319	**0.00744**
CountryNigeria		-0.56440	0.33289	0.08999	0.105
Number_alleles		0.01232	0.01487	0.40717	0.407
ELW		0.07120	0.01417	5.07e-07	**0.00000177**
SexM		-0.39087	0.20452	0.05598	0.0948
ManaMHC_009:Co	untryNigeria	-116,671	0.63863	0.06771	0.0948
**b)**		**Estimate**	**Std. error**	**p-values**	**fdr-corrected**
(Intercept)		-299,709	0.60237	6.51e-07	**0.00000455**
ManaMHC_011		-0.03012	0.36842	0.935	0.935
CountryNigeria		-0.47114	0.35250	0.181	0.314
Number_alleles		0.01790	0.01473	0.224	0.314
ELW		0.06752	0.01421	2.01e-06	**0.00000703**
SexM		-0.41399	0.20262	0.041	0.0957
ManaMHC_011:Co	untryNigeria	0.13277	0.48408	0.784	0.915
**c)**		**Estimate**	**Std. error**	**p-values**	**fdr-corrected**
(Intercept)		-288,251	0.56002	2.64e-07	**0.00000107**
ManaMHC_012		-0.27759	0.29644	0.3491	0.407
CountryNigeria		-0.43678	0.26005	0.0930	0.163
Number_alleles		0.02003	0.01449	0.1670	0.234
ELW		0.06867	0.01341	3.05e-07	**0.00000107**
SexM		-0.42533	0.20294	0.0361	0.0842
ManaMHC_012:Co	untryNigeria	-0.38206	0.47299	0.4192	0.419
**d)**		**Estimate**	**Std. error**	**p-values**	**fdr-corrected**
(Intercept)		-305,950	0.60743	4.74e-07	**0.00000284**
ManaMHC_021		-0.30096	0.34674	0.3854	0.440
CountryNigeria		-0.29369	0.38029	0.4399	0.440
Number_alleles		0.01899	0.01448	0.1897	0.285
ELW		0.06660	0.01416	2.57e-06	**0.00000770**
SexM		-0.40251	0.20304	0.0474	0.0949
**e)**		**Estimate**	**Std. error**	**p-values**	**fdr-corrected**
(Intercept)		-287,990	0.60165	1.70e-06	**0.00000684**

ManaMHC_022		-0.29864	0.27486	0.2772	0.298
CountryNigeria		-0.65339	0.37752	0.0835	0.146
Number_alleles		0.01982	0.01485	0.1821	0.255
ELW		0.06775	0.01424	1.96e-06	**0.00000684**
SexM		-0.41637	0.20285	0.0401	0.0936
ManaMHC_022:Co	untryNigeria	0.43578	0.41856	0.2978	0.298
**f)**		**Estimate**	**Std. error**	**p-values**	**fdr-corrected**
(Intercept)		-302,078	0.58962	3.00e-07	**0.00000180**
ManaMHC_025		-0.04548	0.39449	0.908	0.908
CountryNigeria		-0.43218	0.32884	0.189	0.233
Number_alleles		0.01892	0.01457	0.194	0.233
ELW		0.06780	0.01421	1.82e-06	**0.00000546**
SexM		-0.41173	0.20248	0.042	0.0840
**g)**		**Estimate**	**Std. error**	**p-values**	**fdr-corrected**
(Intercept)		-302,909	0.58788	2.57e-07	**0.00000154**
ManaMHC_027		-0.11074	0.35581	0.7556	0.756
CountryNigeria		-0.42154	0.32385	0.1930	0.232
Number_alleles		0.01945	0.01466	0.1847	0.232
ELW		0.06792	0.01417	1.64e-06	**0.00000493**
SexM		-0.41213	0.20243	0.0418	0.0835
**h)**		**Estimate**	**Std. error**	**p-values**	**fdr-corrected**
(Intercept)		-304,564	0.56856	8.47e-08	**0.000000593**
ManaMHC_033		-214,059	105,818	0.0431	0.0754
CountryNigeria		-0.51561	0.28325	0.0687	0.0962
Number_alleles		0.02353	0.01501	0.1168	0.136
ELW		0.07177	0.01579	5.51e-06	**0.0000193**
SexM		-0.47281	0.20352	0.0202	**0.0471**
ManaMHC_033:Co	untryNigeria	158,178	111,658	0.1566	0.157
**i)**		**Estimate**	**Std. error**	**p-values**	**fdr-corrected**
(Intercept)		-295,394	0.59119	5.84e-07	**0.00000341**
ManaMHC_050		0.42086	0.33993	0.2157	0.259
CountryNigeria		-0.56343	0.33950	0.0970	0.145
Number_alleles		0.01628	0.01463	0.2660	0.266
ELW		0.06868	0.01411	1.14e-06	**0.00000341**
SexM		-0.41771	0.20271	0.0393	0.0787

**j)**		**Estimate**	**Std. error**	**p-values**	**fdr-corrected**
(Intercept)		-286,628	0.60440	2.11e-06	0.0000105
ManaMHC_069		-0.81493	0.33307	0.0144	**0.0336**
CountryNigeria		-0.66130	0.35210	0.0604	0.0704
Number_alleles		0.02012	0.01452	0.1659	0.166
ELW		0.06600	0.01413	2.99e-06	**0.0000105**
SexM		-0.39865	0.20434	0.0511	0.0704
ManaMHC_069:Co	untryNigeria	118,062	0.51011	0.0206	**0.0361**
**k)**		**Estimate**	**Std. error**	**p-values**	**fdr-corrected**
(Intercept)		-297,719	0.58650	3.85e-07	**0.00000231**
ManaMHC_104		0.37491	0.37419	0.3164	0.316
CountryNigeria		-0.52776	0.33739	0.1178	0.177
Number_alleles		0.01748	0.01456	0.2300	0.276
ELW		0.06847	0.01431	1.71e-06	**0.00000513**
SexM		-0.41760	0.20271	0.0394	0.0788
**l)**		**Estimate**	**Std. error**	**p-values**	**fdr-corrected**
(Intercept)		-289,424	0.57392	4.58e-07	**0.00000160**
ManaMHC_107		0.44738	0.42047	0.2873	0.402
CountryNigeria		-0.60423	0.29152	0.0382	0.0891
Number_alleles		0.01207	0.01492	0.4188	0.426
ELW		0.07192	0.01390	2.27e-07	**0.00000159**
SexM		-0.38949	0.20329	0.0554	0.0969
ManaMHC_107:Co	untryNigeria	0.48163	0.60557	0.4264	0.426
**m)**		**Estimate**	**Std. error**	**p-values**	**fdr-corrected**
(Intercept)		-288,805	0.56909	3.88e-07	**0.00000116**
ManaMHC_197		0.68011	0.36994	0.0660	0.0792
CountryNigeria		-0.68135	0.27938	0.0147	**0.0295**
Number_alleles		0.01515	0.01469	0.3026	0.303
ELW		0.07100	0.01376	2.47e-07	**0.00000116**
SexM		-0.42731	0.20272	0.0350	0.0526
