# Supplementary material for: Regional Lassa virus lineages select for divergent MHC-I repertoires in Mastomys natalensis rodents
Source: PLoS Pathog. 2026 Apr 17;22(4):e1014121. doi: 10.1371/journal.ppat.1014121 (PMC13124061; doi:10.1371/journal.ppat.1014121)
Supplement: S5 Table — (PDF) [file ppat.1014121.s009.pdf]

S5 Table. Generalised linear mixed effect model results for the effect of MHC supertypes, host sex, country and eye lens weight on LASV detection.

**a)**	**Estimate**	**Std. Error**	**p-value**
(Intercept)	*-2,19404	0.48023	4.91e-06
Supertype_5	0.43427	0.35928	0.227
CountryNigeria	*-1,00679	0.66130	0.128
ELW	0.00172	0.01702	0.919
SexM	*-0,08669	0.25801	0.737
Supertype_5:CountryNigeria	0.76097	0.72055	0.291
**b)**	**Estimate**	**Std. Error**	**p-value**
(Intercept)	*-4,209071	1,313,277	0.00135
Supertype_18	2	1,036,433	0.06671
CountryNigeria	2	1,100,501	0.16416
Number_ST	0.033964	0.060745	0.57607
ELW	0.004113	0.016994	0.80876
SexM	*-0,086033	0.259037	0.73979
Supertype_18:CountryNigeria	*-1,992636	1,134,563	0.07904
