# Supplementary material for: Regional Lassa virus lineages select for divergent MHC-I repertoires in Mastomys natalensis rodents
Source: PLoS Pathog. 2026 Apr 17;22(4):e1014121. doi: 10.1371/journal.ppat.1014121 (PMC13124061; doi:10.1371/journal.ppat.1014121)
Supplement: S6 Table — (PDF) [file ppat.1014121.s010.pdf]

S6 Table. Generalised linear mixed effect model results for the effect of MHC supertypes, host sex, country and eye lens weight on IgG detection.

**a)**	**Estimate**	**Std. Error**	**p-value**	**fdr-corrected**
(Intercept)	-3,02738	0.74227	4.53e-05	<0.001
Supertype_15	-0,83406	0.27919	0.00281	**0.00562**
CountryNigeria	-1,00958	0.40706	0.01313	0.02626
Number_ST	0.07192	0.04556	0.11447	0.22894
ELW	0.07051	0.01407	5.41e-07	**<0.001**
SexM	-0,43580	0.20457	0.03314	0.06628
Supertype_15:CountryNigeria	0.87123	0.42982	0.04267	0.08534
**b)**	**Estimate**	**Std. Error**	**p-value**	**fdr-corrected**
(Intercept)	-3,22905	0.76292	2.31e-05	<0.001
Supertype_5	0.64588	0.29840	0.0304	**0.03040**
CountryNigeria	-0,09478	0.46008	0.8368	0.83680
Number_ST	0.02498	0.04837	0.6056	0.6056
ELW	0.06963	0.01427	1.06e-06	**<0.001**
SexM	-0,41561	0.20350	0.0411	0.06628
Supertype_5:CountryNigeria	-0,65849	0.45398	0.1469	0.14690
