# Supplementary material for: Regional Lassa virus lineages select for divergent MHC-I repertoires in Mastomys natalensis rodents
Source: PLoS Pathog. 2026 Apr 17;22(4):e1014121. doi: 10.1371/journal.ppat.1014121 (PMC13124061; doi:10.1371/journal.ppat.1014121)
Supplement: S7 Table — (PDF) [file ppat.1014121.s011.pdf]

S7 Table. Other RNA viruses detected in LASV-positive *Mastomys natalensis* individuals in localities endemic for LASV lineage II within Nigeria

RNA virus	Locality	Number of individuals
Picornaviruses		
	Ebudin	7
	Ekpoma	1
	Okeluse	1
Hepaciviruses		
	Ebudin	3
